# The incubation period of hepatitis E genotype 1: insights from pooled analyses of travellers

**DOI:** 10.1017/S0950268818001097

**Published:** 2018-05-24

**Authors:** A. S. Azman, I. Ciglenecki, C. Oeser, B. Said, R. S. Tedder, S. Ijaz

**Affiliations:** 1 Department of Epidemiology, Johns Hopkins Bloomberg School of Public Health, Baltimore, Maryland, USA; 2 Médecins sans Frontières, Geneva, Switzerland; 3 Emerging Infections and Zoonoses, National Infection Service, Public Health England, London, UK; 4 Blood Borne Virus Unit, National Infection Service, Public Health England, London, UK; 5 Division of Infection and Immunity, University College London, London, UK; 6 Transfusion Microbiology, NHS Blood and Transplant, London, UK

**Keywords:** Hepatitis E, HEV, incubation period, survival analysis, travellers

## Abstract

Hepatitis E virus genotype 1 (HEV G1) is an important cause of morbidity and mortality in Africa and Asia. HEV G1's natural history, including the incubation period, remains poorly understood, hindering surveillance efforts and effective control. Using individual-level data from 85 travel-related HEV G1 cases in England and Wales, we estimate the incubation period distribution using survival analysis methods, which allow for appropriate inference when only time ranges, rather than exact times are known for the exposure to HEV and symptom onset. We estimated a 29.8-day (95% confidence interval (CI) 24.1–36.0) median incubation period with 5% of people expected to develop symptoms within 14.3 days (95% CI 10.1–21.7) and 95% within 61.9 days (95% CI 47.4–74.4) of exposure. These estimates can help refine clinical case definitions and inform the design of disease burden and intervention studies.

## Background

Hepatitis E virus genotypes 1 and 2 (HEV G1 and HEV G2) are an important cause of acute viral hepatitis E and jaundice worldwide and are thought to be responsible for more than 50 000 deaths annually, primarily in Africa and Asia [1]. HEV G1 and G2 are known to be responsible for outbreaks and thought to spread primarily from person-to-person by faecal contamination of food and water [2, 3]. In recent years, outbreaks, typically linked to HEV G1, have been regularly identified in camps for displaced persons in Africa resulting in substantial morbidity and mortality [4, 5]. Controlling epidemic hepatitis E has been challenging to the public health community due to the lack of readily available efficacious tools and poor understanding of the epidemiology of the disease [6, 7].

The incubation period of an infectious disease, defined as the time from exposure to a pathogen to the onset of symptoms, plays an important role in clinical and public health decision-making [8, 9]. For hepatitis E, our understanding of this key property is based on individual case reports, with no published quantitative synthesis of the full distribution of the incubation period. In general, statements about the incubation period of hepatitis E suggest a range from 2 to 10 weeks and a mean of 25–50 days. Whether the incubation period differs between G1/G2 compared with other genotypes (e.g. G3/G4) as a result of different transmission routes remains unknown [3, 10–12].

While the mean (or median) of the incubation period may be useful for summarisation, the tails of the incubation period distribution (e.g. the 5th and 95th percentiles) play an important role in public health and clinical practice alike. In clinical practice, understanding the range of plausible time delays between exposure and symptom onset can help clinicians assess the likelihood that an acute jaundice case with recent travel to a hepatitis E endemic area could be caused by HEV infection. In epidemiologic studies and in outbreak response, understanding the relevant period for putative exposures is a key to better understanding hepatitis E transmission and improving control efforts.

Travellers from countries where the risk of HEV G1 infection is thought to be close to zero, who return from countries known to have hepatitis E and present subsequently with acute jaundice, provide a unique opportunity to learn about the natural history of the disease, including the incubation period. Here we analyse data from patients with confirmed acute hepatitis E in England and Wales with known recent travel to hepatitis E endemic areas and estimate the full distribution of the incubation period providing new quantitative insights into the natural history of this disease.

## Methods

### Study population and laboratory methods

As part of the Public Health England (PHE) programme of enhanced surveillance for HEV infections, virological, demographic, and where possible, clinical, risk and travel history information on diagnosed acute hepatitis E cases are collated. Two PHE reference laboratories, one based in North London and one in Birmingham, carry out most primary diagnostic testing for HEV in England and Wales. Suspected cases are identified by health professionals based on the signs or symptoms of hepatitis E including clinically apparent jaundice or abnormal liver transaminases. Plasma/serum samples taken at the time of presentation from suspected cases are sent from microbiology laboratories for HEV marker testing. Virological testing of HEV markers includes the detection of IgM and IgG antibodies to HEV (anti-HEV) using the Wantai assays (Fortress Diagnostics, Northern Ireland) and HEV RNA detection and quantification carried out as previously described [13]. Sequencing, genotypic and phylogenetic analysis across part of the ORF2 is undertaken on all HEV RNA-positive samples (usually only possible with a viral load >5000 IU/ml) as previously described [14]. A confirmed acute hepatitis E case is based on the detection of HEV IgM and/or RNA.

Upon laboratory confirmation, cases are contacted directly by public health professionals, and using a structured questionnaire, information on travel history including place and dates of travel and onset of symptoms are collected. Cases are considered ‘travel-related’ where the patient has returned within 9 weeks from a country thought to have ongoing HEV G1 or G2 transmission.

Based on virological, epidemiological and travel information collated from testing at the London PHE laboratory, 85 confirmed acute hepatitis E cases with sample collection dates between 31 January 2011 and 21 September 2016 were included in these analyses.

### Statistical methods

The primary data in these analyses consist of dates of travel and time range of symptom onset. We treated these data as doubly interval censored, allowing for both the exposure and the symptom onset periods to be ranges rather than specific dates. We used previously published parametric survival analysis methods to estimate the parameters of the incubation period distribution [15, 16]. These methods do not require us to assign a specific date of exposure to each person, rather they assign a uniform probability of exposure to each day of a person's travel. Incubation periods have traditionally been thought to follow log-normal distributions characterised by a mean and dispersion, with 2/3 of individual incubation periods falling between mean/dispersion and the mean  ×  dispersion [8]. Following this convention, we fit the data to log-normal distributions. We also considered gamma, Erlang and Weibull distributed incubation periods and compared support for the different models with the log likelihood. We fit models using a maximum-likelihood framework with the coarseDataTools package (version 0.6-3, [17]) with the R statistical language (R Foundation, Vienna, Austria). From these analyses, we estimated the times where 1, 5, 25, 50, 75, 95 and 99% of individuals are expected to develop symptoms after exposure to HEV G1. We used the 2.5th and 97.5th percentiles of estimates based 2500 bootstrapped datasets as the 95% confidence intervals (CIs).

## Results

Most cases were in the mid-acute/early-recovery phase of HEV infection at the time of sample collection, with 83 of 85 patients having samples that were anti-HEV IgM-positive, IgG-positive and RNA-positive or anti-IgM- and IgG-positive but RNA-negative. Two patients had samples that were IgM- and RNA-positive but IgG-negative, indicating an early acute HEV infection. HEV genotyping was possible in samples from 52 (61%) patients and all were found to be HEV G1 viruses.

Most cases reported travel to a single country but some reported travel to two within the relevant time window. Cases reported travel to India (51/85), Pakistan (21/85), Bangladesh (6), Egypt (3), Afghanistan (1), Saudi Arabia (1), The Philippines (person also visited India during trip) and South Sudan (1). Data on the travel location for one case was not available. Cases ranged in age from 13 to 81 years with a median age of 42 (interquartile range (IQR) 30–58) and 33% (15/51) were female.

The median time from self-reported symptom onset to sample collection was 12 days (IQR 7–26, *n* = 52) for those able to be genotyped and 19 days (IQR 8–33, *n* = 33) for those unable to be genotyped (likely those clearing the virus with lower viral load). At the time of testing, 98% (83 of 85) individuals were anti-HEV IgG sero-positive. All individuals tested positive for anti-HEV IgM.

**Fig. 1.**
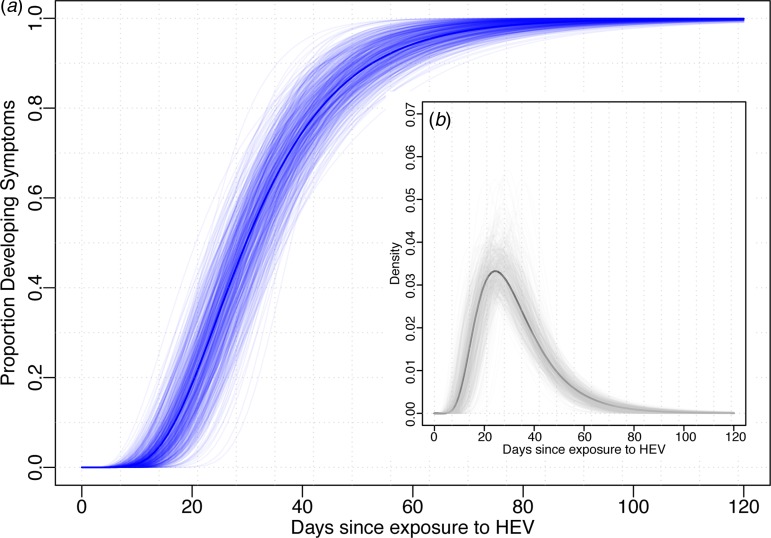
Incubation period distribution (log-normal model). (a) Shows the cumulative distribution of the incubation period (dark blue line) with bootstraps in light blue lines. (b) Shows the density function of the incubation period (black) with bootstrap estimates in light grey. Estimates of quantiles for this model are in the first row of Table 1.

Using data from the 52 cases confirmed to have HEV G1, we estimated the median incubation period to be 29.8 days (95% CI 24.1–36.0) with a dispersion of 1.6 (95% CI 1.3–1.8). Thus, 5% of individuals are expected to develop symptoms within 14.3 days (95% CI 10.1–21.7), 25% within 22.0 (95% CI 17.1–29.0), 75% within 40.2 (95% CI 32.8–46.8) and 95% within 61.9 (95% CI 47.4–74.4) days after exposure (Fig. 1). The first 1% will develop symptoms within 10.6 days (95% CI 7.0–17.9) and the last 1% after more than 83.8 (95% 58.5–106.5) days post-exposure. While these estimates are based on the conventional log-normal model, alternative parametric models yielded similar results (Table 1). As the non-genotyped cases were suspected hepatitis E infections linked to travel to areas known to have HEV G1 cases, we also combined all data (*n* = 85) to estimate a slightly short median incubation period of 26.4 days (95% CI 21.5–31.5) days (Table 1).

**Table 1.** Comparison of incubation period estimates for alternative parametric models and datasets

Model	5th percentile	25th percentile	50th percentile	75th percentile	95th percentile	Shape	Scale	−2 log likelihood
Log-normal	14.3 (10.1–21.7)	22.0 (17.1–29.0)	29.8 (24.1–36.0)	40.2 (32.8–46.8)	61.9 (47.4–74.4)	3.4 (3.2–3.6)	0.45 (0.27–0.57)	−22.7
Gamma	13.2 (8.6–20.5)	22.3 (17.4–29.0)	30.9 (25.2–37.1)	41.3 (33.8–48.2)	60.1 (45.7–71.5)	5.1 (3.1–13.4)	6.5 (2.4–10.4)	−23.6
Weibull	11.2 (6.9–19.6)	22.6 (17.1–30.2)	32.4 (26.0–39.0)	42.9 (34.5–49.9)	58.7 (44.3–69.8)	2.5 (1.9–4.4)	37.6 (30.6–44.1)	−23.7
Erlang^a^	13.3 (8.3–17.6)	22.3 (17.1–27.3)	30.7 (25.5–36.8)	41.2 (34.7–49.5)	60.1 (49.6–75.6)	5.0 (3.0–8.0)	6.4 (4.0–11.7)	^a^
Full data^b^	10.35 (7.0–16.5)	18.0 (13.6–24.1)	26.4 (21.5–24.1)	38.8 (32.9–44.8)	67.5 (53.3–81.2)	3.3 (3.1–3.5)	0.57 (0.38–0.72)	

a Fit using Bayesian version of the doubly interval censored data as implemented in *coarseDataTools* package in R with default priors and 10 000 samples after a burn-in of 5000.

b Log-normal model fit to data from both G1-positive (genotyped) samples and those not able to be genotyped.

First four rows represent models fit to only genotyped cases and final row represents model fit to all data including those without genotype results available. Top row represents the model presented in the main analyses.

## Discussion

Using a national dataset of travel-related cases in England and Wales, we estimated the full incubation period distribution of HEV G1. We found that the median incubation period (30 days) is consistent with general references in the literature to hepatitis E, although the upper and lower tails of the incubation period distribution are more extreme than generally mentioned, potentially reflecting the heterogeneity in the human response to the virus, the virus itself or the inoculum size. This characterisation of the incubation period distribution has implications for decision-making in both the clinical and public health domains.

The tails of the incubation period distribution can serve as a guide for optimal time windows in which to consider potential exposures related to hepatitis E. For surveillance systems aiming to capture all travel-related cases, it may be advisable to consider all travel between 10 and 75 days before symptom onset to achieve high sensitivity. This plays a role in clinical triaging as well as epidemiologic studies, including those trying to better understand the routes of transmission and risk factors for the disease. In case–control studies during or after outbreaks, a similar time window may be desired, although it must be balanced with the likelihood of differential recall bias as the time window grows and the desire for higher specificity. While our estimates focus on HEV G1, incubation periods inferred from point-source foodborne outbreaks in Europe of HEV G3 are consistent with our estimates [18]. Our estimates may be particularly relevant to inform the design and analysis of future observational studies on the effectiveness of hepatitis E vaccine. Finally, these estimates can serve as a reference for computational models of hepatitis E transmission, which normally require strong assumptions about the incubation period distribution.

This study comes with several limitations. The patients included in our study represent only those suspected to have hepatitis E by their care providers, thus these may represent only the more severe and clinically apparent cases of HEV infection. Severity could be related to the duration of the incubation period, with more severe cases having shorter incubation periods than those with mild disease, although little evidence specific to HEV exists to support or refute this possibility. Our sample represents travellers, whose baseline health may on average be better to those living in the populations where hepatitis E G1 transmission tends to occur, which also may be related to the incubation period duration. Finally, since the time since travel (a maximum of 9 weeks) was used in classifying suspected cases as having been travel-related or not, this may have biased our estimates of the incubation period downwards through excluding longer incubation periods.

Hepatitis E genotype 1 remains an important cause of morbidity and mortality, particularly in developing countries in Africa and Asia. As control of epidemics continues to pose challenges [3–5], new insights into the key aspects of dynamics of this disease, including the incubation period, may shed light on more effective control and prevention strategies.
